# Potentiated serotonin signaling in serotonin re‐uptake transporter knockout mice increases enterocyte mass and small intestinal absorptive function

**DOI:** 10.14814/phy2.14278

**Published:** 2019-11-14

**Authors:** Chasen J. Greig, Lucy Zhang, Robert A. Cowles

**Affiliations:** ^1^ Section of Pediatric Surgery Department of Surgery Yale School of Medicine New Haven Connecticut

**Keywords:** Absorptive function, citrulline, intestinal mucosal growth, mouse model

## Abstract

Genetic knockout of the serotonin reuptake transporter (SERT) potentiates serotonin signaling and increases crypt‐cell proliferation, neuroplasticity, and mucosal surface area. However, it remains unknown whether these changes occur throughout the small intestine and whether they increase nutrient absorption. We hypothesized that serotonin‐mediated mucosal growth would occur throughout the intestine and would increase enterocyte mass and absorptive function. Following institutional approval, intestinal segments spanning the bowel were harvested from 10 to 12 week‐old SERT knockout (SERTKO) and wild‐type (WT) C57Bl/6 mice. Histologic sections were used to measure villus height (VH), crypt depth (CD), and crypt proliferation index (CPI). Plasma citrulline was measured colorimetrically. Glucose and peptide absorption in isolated segments of small bowel were calculated using a previously described method for quantification after luminal instillation of substrate. At baseline, morphometric (VH/CD) and proliferative (CPI) parameters varied from jejunum to ileum. Enhanced 5‐HT signaling significantly increased plasma citrulline levels and morphometric/proliferative parameters in all regions analyzed. Glucose absorption in WT mice varied throughout the small intestine, and SERTKO mice demonstrated significant increases in the middle and distal bowel. WT peptide absorption was similar throughout the small bowel, and SERTKO mice had significant increases in the proximal and distal bowel. Enhanced serotonin signaling results in increased morphometric and proliferative parameters throughout the small intestine, and results in increased enterocyte mass and intestinal absorptive function. These data further advance the concept that the serotonin system is an attractive therapeutic target for increasing functional intestinal mucosa.

## Graphical abstract

Enhanced serotonin signaling in the murine enteric nervous system stimulates mucosal growth throughout the small bowel, but most significantly in its distal segments. Importantly, these anatomic changes are associated with evidence for greater enterocyte mass that translates into increased glucose and peptide absorption. Taken together, these findings suggest enteric serotonin acts as a mucosal growth factor supporting the concept of enteric serotonin signaling as a potential therapeutic target.

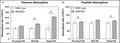

## Introduction

Short bowel syndrome (SBS) is the most common cause of intestinal failure (IF) and is responsible for significant morbidity and mortality (Squires et al. 2012). Currently, the management of SBS relies upon parenteral nutrition (PN) and promotion of intestinal adaptation, although the surgical management of SBS has undergone an extensive maturation process in the last several decades (Bines 2009; Sommovilla and Warner 2014). Despite advances in our ability to provide safe PN and innovations in the area of surgical lengthening procedures, few therapies aimed at adding functional intestinal mucosa currently exist. For these reasons, a broader understanding of the complex processes underlying small intestinal mucosal growth is essential in order to guide development of desperately needed therapies for this morbid condition. Recent investigations into the role of serotonin as an intestinal growth factor suggest serotonin signaling holds potential as a therapeutic target for increasing small intestinal mucosal function.

Enteric serotonin (5‐HT) is the most abundant neurotransmitter in the intestine and has been implicated in a wide range of physiologic processes in the gastrointestinal tract (Bornstein 2012; Gershon 2013). While most commonly thought of for its critical role in the central nervous system, 95% of the 5‐HT found in the body resides in the gastrointestinal tract and functions within the mucosa and enteric nervous system (Gershon and Tack 2007).

Our laboratory has previously studied enhanced 5‐HT signaling in the murine intestine using a serotonin re‐uptake transporter knockout (SERTKO) mouse model. Previous data using this model have shown enhanced mucosal morphometric parameters (villus height and crypt depth) resulting in increased mucosal surface area as well as increased crypt cell proliferation and neuroplasticity in the ileum, all while maintaining normal physiologic cellular composition of the ileal mucosa, including the number of 5‐HT‐producing enterochromaffin cells (Gross et al. 2012; Greig et al. 2016; Tackett et al. 2017; Greig and Cowles 2017a). While these data held promise in the investigation of the 5‐HT system as a therapeutic target for increasing functional mucosa, it remains unknown whether the mucosal growth stimulated by 5‐HT would occur throughout the small intestine, and whether these changes would result in enhanced intestinal absorptive function. Thus, we aimed to determine the effect of 5‐HT‐mediated mucosal growth on small intestinal absorptive capacity in the murine small intestine. We hypothesized that SERTKO animals would demonstrate increased mucosal and proliferative parameters when compared to wild‐type (WT) mice, as demonstrated previously, and that these changes would result in increased enterocyte mass and absorptive function.

## Methods

### Ethical approval

Experiments in this study were approved by Yale University’s IACUC (protocol #11567). Yale University’s IACUC is governed by applicable Federal and State regulations, including those of the Animal Welfare Act (AWA), Public Health Service (PHS), and the United States Department of Agriculture (USDA) and is guided by the U.S. Government Principles for the Utilization and Care of Vertebrate Animals Used in Testing, Research and Training.

### Animals

The serotonin reuptake transporter (SERT) molecule is a high‐affinity transporter of 5‐HT and responsible for its signaling inactivation by way of intracellular transport. SERT knockout mouse models have been generated by deletion of this gene, which results in decreased signaling inactivation of 5‐HT and thus enhanced 5‐HT effects. Wild‐type (WT) and SERT knockout (SERTKO) mice were bred on a C57BL/6 background at the Yale Animal Resource Facility at Yale School of Medicine. All animals were housed in a pathogen‐free environment with a 12‐h light/dark cycle with food and water ad libitum. 10‐ to 12‐week‐old male mice were used for all experiments. Animal weights were recorded at the start of the experiments.

### Anesthetic administration and euthanasia

Fasting mice received inhalational anesthesia (isoflurane 2–3% induction, 0.25–2% maintenance – inhaled). Depth of anesthesia was monitored by monitoring of respiratory rate and by toe‐pinch looking for reaction from the mouse every 5 min during the procedure. Euthanasia was performed using CO_2_ asphyxiation followed by confirmation of death with cervical dislocation.

### Histologic measurements and immunohistochemistry

Using six mice from each group (WT and SERTKO), 2 cm segments from the proximal jejunum, distal jejunum, proximal ileum, and distal ileum were procured and fixed in 10% Neutral Buffered Formalin. These were then paraffin‐embedded and histologic sections created. Standard immunohistochemical protocols were used to create H&E and chromogenic slides for measurements of morphometric and proliferative parameters, respectively. Examiners were blinded to the samples at the time of measurements.

For chromogenic slides used to calculate crypt proliferation index (CPI), a mouse monoclonal primary antibody against Ki67 at 1:250 dilution (Cell Signaling Technology, Danvers, MA) followed by DAB substrate (Thermo Scientific, Waltham, MA) was used as per manufacturer’s protocols and counterstained with hematoxylin. CPI was then calculated as the percentage of Ki67‐positive cells within the total number of crypt cells. Crypts were counted if a single epithelial cell layer was present from crypt‐villus junction to crypt‐villus junction, and at least a portion of the adjacent villi were present. At least 10 crypts per group were counted, and used to calculate the mean CPI.

Morphometric parameters included villus height (VH) and crypt depth (CD). At least 20 villi and crypts were measured per group. Villi were chosen for measurement when intact from crypt‐villus junction to crypt‐villus junction and central lacteal present. Crypts were measured when intact from crypt‐villus junction to crypt‐villus junction and at least partial visualization of adjacent villi present.

### Measurement of serum citrulline levels

Serum citrulline is a known and validated biomarker for enterocyte mass (Crenn et al. 2008; Gutierrez et al. 2014). Serum samples were collected from six mice from each group using the saphenous method, similar to previously described (Patel et al. 2016). Briefly, mice were anesthetized in a warmed and humidified isolette, and 100 *µ*L samples were collected and placed in BD serum separator microtainers (Becton‐Dickenson, Franklin Lakes, NJ) for isolation of serum for analysis. Citrulline was measured using a commercially available colorimetric assay kit (Cell Biolabs, Inc., San Diego, CA).

### Glucose absorption

Glucose absorption in isolated and perfused intestinal segments was measured, as previously described (Sullins et al. 2014). Briefly, three mice from each group were fasted overnight prior to induction of anesthesia and laparotomy in a warmed and humidified isolette. The small bowel was identified from the ligament of Trietz to the ileocecal valve and three 2 cm segments (distal ileum, mid small bowel and proximal jejunum) were isolated by application of suture tied to occlude the bowel lumen. A mixture of glucose and phenol red was then injected into each segment and, after 15 min, all liquid was aspirated. The phenol red is a non‐absorbable compound used to correct for changes in water content when assessing glucose absorption, and previously used for similar purposes (Tugcu‐Demiroz et al. 2014). The glucose content of the fluid was quantified colorimetrically via a commercially available assay kit (MilliporeSigma, Burlington, MA). Changes in glucose concentration were then calculated as a function of time and length of small bowel.

### Peptide absorption

D‐alanine‐lysine‐N‐7‐amino‐4‐methylcoumarin‐3‐acetic acid (D‐Ala‐Lys‐AMCA; Sigma‐Aldrich, St. Louis, MO) is a fluorophore‐conjugated peptide molecule used previously as a reporter molecule for peptide transport (Otto et al. 1996; Groneberg et al. 2001). Using the method described above for glucose absorption, we used D‐Ala‐Lys‐AMCA to determine peptide absorption in perfused, isolated segments of intestine. Fluorescence of the luminal aspirate was quantified after correction for fluid shifts.

### Statistical analysis

Statistical analysis was performed using Prism Software (GraphPad, San Diego, CA). Groups were compared using Student’s t‐test with *P* < 0.05 indicating significance.

## Results

### Mucosal growth and proliferation

**Figure 1:**
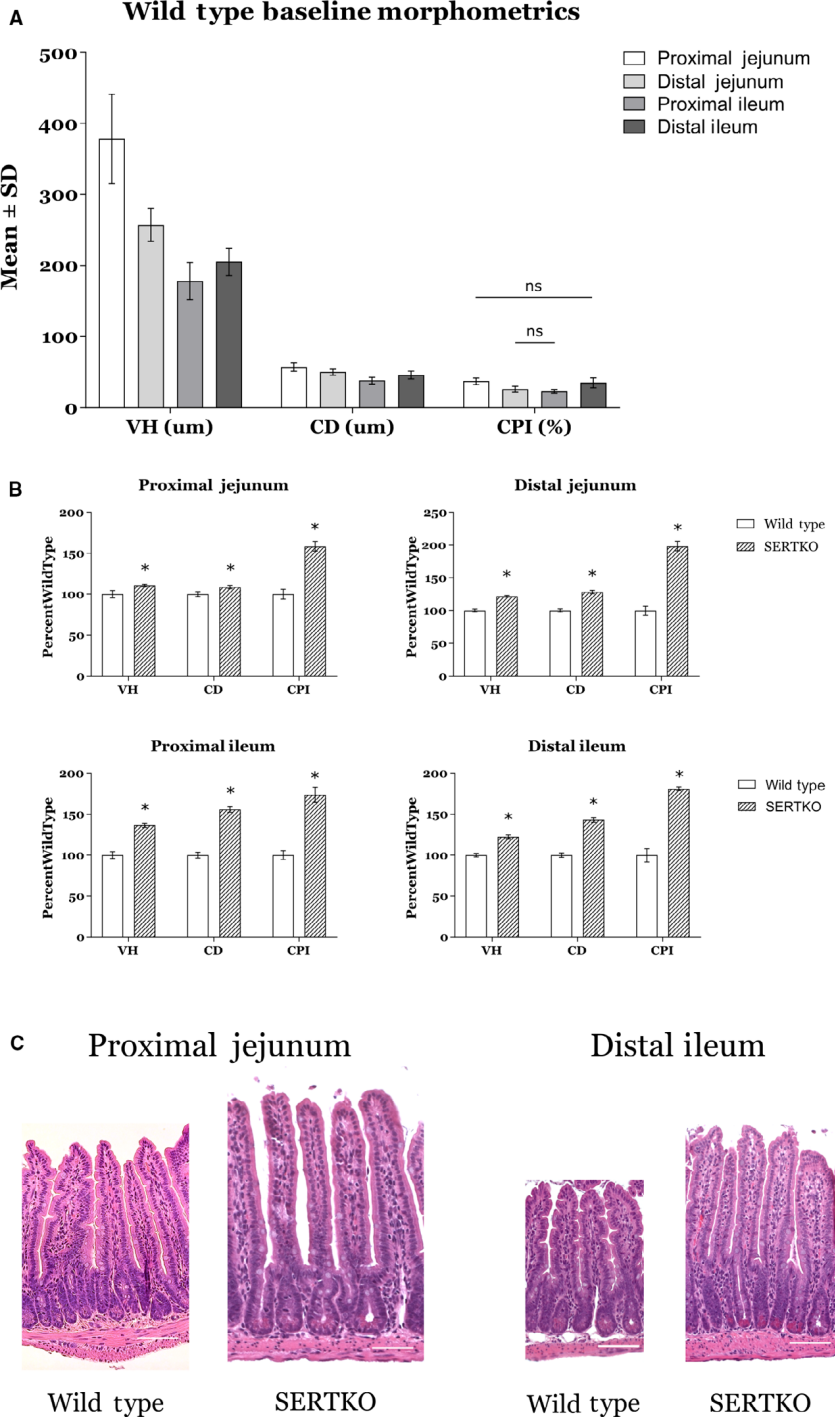
(A) Baseline morphometric and proliferative parameters for wild‐type animals varied throughout the small bowel. In general, there were taller villi and deeper crypts present in the proximal small bowel compared to the distal small bowel. Nearly all regions for each parameter were significantly different from each other (*P* < 0.05), with the exception of proximal jejunum/distal ileum and distal jejunum/proximal ileum for CPI (ns = not significant). (B) SERTKO animals had taller villi, deeper crypts, and increased crypt cell proliferation in all regions of the small bowel analyzed compared with wild‐type animals. The most significant increases in both morphometric and proliferative parameters were seen in the proximal and distal ileum of SERTKO animals (*P < 0.05). (C) Representative H&E images at 200X magnification of proximal and distal small bowel sections from wild‐type and SERTKO animals. Scale bar: 50 *µ*m. All data shown as mean ± SD. VH = villus height, CD = crypt depth, CPI = crypt proliferation index.

Starting weights did not significantly differ between experimental groups, and mean ± SD weights for WT were 21.6 ± 3.2 g and SERTKO were 20.0 ± 3.8 g. At baseline in wild‐type animals, morphometric (VH and CD) and proliferative (CPI) parameters varied throughout the small bowel, with a trend toward decreased morphometric and proliferative parameters moving from proximal to distal bowel (Fig. 1A). When parameters were compared between WT and SERTKO animals, there were significant differences for both morphometric and proliferative parameters in all areas of the intestine analyzed (Fig. 1B). The largest differences for both morphometric and proliferative parameters were seen in the proximal and distal ileum (Fig. 1B).

### Serum citrulline levels

**Figure 2:**
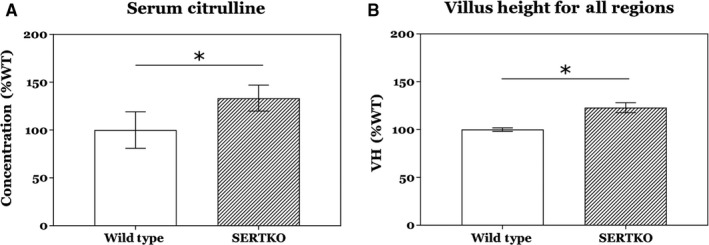
(A) Serum citrulline levels, a putative marker for enterocyte mass, were significantly increased by approximately 33% in SERTKO animals compared to wild type. (B) As expected, the composite villus height measurements for all regions of the intestine were increased by approximately 23% in SERTKO animals compared to wild type, commensurate with the increase seen in citrulline levels. All data shown as mean ± SD. *P < 0.05.

Serum citrulline levels were significantly higher in SERTKO animals compared to WT, with the mean levels approximately 33% higher in SERTKO animals (Fig. 2A). When compared to our morphometric data, these data are in general agreement with our results for aggregate VH averaged across all areas analyzed, with a mean overall increase in VH of approximately 23% for SERTKO animals (Fig. 2B).

### Glucose and peptide absorption

**Figure 3:**
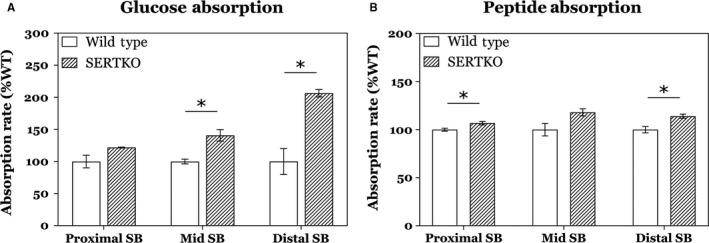
Intestinal absorptive function was significantly increased in SERTKO animals compared to wild type. (A) Glucose absorption was increased in all regions of the small bowel analyzed, with significant increases in the mid and distal small bowel. (B) Peptide absorption was increased in all regions of the small bowel, with statistically significant increases in the proximal and distal bowel. Overall, these data correlate with our anatomic data, which showed the largest increases in the distal small bowel. All data shown as mean ± SD. *P < 0.05.

Absorption studies demonstrated increased absorption throughout the small bowel for both glucose and peptide. These increases were statistically significant in the mid and distal small bowel for glucose absorption, while peptide absorption had statistically significant increases in the proximal and distal small bowel (Fig. 3). Both experiments had significant increases in the distal bowel, and glucose absorption was largest in this area of the bowel by a large margin. These data are also in agreement with our morphometric findings that the largest increases in morphometric and proliferative parameters in SERTKO animals occurred in the distal bowel.

## Discussion

SBS is the most common cause of intestinal failure and remains a highly morbid condition despite major advancements in treatment including improvements in parenteral nutrition (Bines 2009; Squires et al. 2012; Sommovilla and Warner 2014). The current management strategies are primarily aimed at fostering intestinal adaptation, but novel therapies that increase the number of functioning enterocytes are lacking and are critically needed.

Serotonin signaling in the small intestine has been extensively studied, but much of the focus has been related to the role in intestinal motility and more recently in inflammatory states of the GI tract (Gershon 2004; Spohn and Mawe 2017). Interestingly, the source of 5‐HT responsible for morphometric growth appears to be neuronal, despite the majority of enteral 5‐HT being found in enterochromaffin cells (Gross et al. 2012). Furthermore, enteric serotonergic neurons do not have known projections to the small intestinal mucosa (Furness and Costa 1982; Meedeniya et al. 1998), and our laboratory hypothesizes that enteric serotonin stimulates the observed mucosal changes through interaction with muscarinic cholinergic neurons in the small bowel mucosa (Greig and Cowles 2017b). We have also previously demonstrated ordered mucosal growth and increased neuroplasticity in the setting of enhanced serotonin signaling in the murine intestine (Greig et al. 2016; Tackett et al. 2017). Taken together, we interpret these data to implicate neuronal serotonin in the small intestine as a mediator of intestinal mucosal growth, resulting in increased morphometric parameters and neuronal projections while maintaining normal cellular architecture. This complex microenvironment and the associated pathways require further study, but initial data suggest enteric serotonin signaling is viable therapeutic target for optimizing intestinal mucosal growth and function.

Our laboratory has studied the effects of serotonin in the small intestine using both a pharmacologic and genetic knockout model in mice. Our pharmacologic model involves administration of selective serotonin reuptake inhibitors (SSRIs), while our genetic knockout model deletes the same SERT gene targeted by SSRIs. Most of the intestinal effects of SSRIs relate to their effect on GI motility. The effect of genetic deletion of SERT in our SERT knockout mice was initially concerning for possible developmental issues, but no major defects were observed and survival was not impacted (Bengel et al. 1998). There are however several known phenotypic changes in these mice, including increased anxiety and reduced aggression (Holmes et al. 2003), but these are not considered severe and, the alterations in the gastrointestinal tract are felt to be minor (Bischoff et al. 2009).

Here we investigated whether 5‐HT‐mediated mucosal growth would result in increased enterocyte mass and mucosal absorptive function. We hypothesized that increases in intestinal mucosal growth in SERTKO mice previously observed in the distal small intestine would occur throughout the small bowel, and that this would logically result in increases in enterocyte mass and in mucosal absorptive function.

We found that at baseline, WT morphometric and proliferative parameters vary throughout the small bowel, with the largest measurements occurring in the proximal small bowel and the lowest in the distal bowel. When we compared morphometric and proliferative parameters of SERTKO animals to WT, we found significant increases in all measured parameters throughout all areas of the bowel. This is in agreement with previous findings from our laboratory, although our previous work did not find a difference in the proximal small bowel (Park et al. 2019). While there are several potential explanations for this discrepancy between those findings and the current results, it is likely related to differences between study designs including the use of a larger age range, both sexes, and harvesting only 3 regions of bowel in the Park, et al. study. Additionally, an increase in mucosal parameters was observed in that study, but did not reach statistical significance as in the current study.

Interestingly, the largest increases were seen in the distal small bowel of SERTKO mice, and this may be explained by the parameters observed in WT mice. Given that the villi and crypts in the proximal bowel of WT mice are large at baseline, the capacity for enhanced 5‐HT signaling to increase these parameters is presumably less. Conversely, enhanced 5‐HT signaling results in substantial increases in the distal bowel, where WT parameters are low at baseline. These data support our hypothesis that enhanced 5‐HT signaling stimulates mucosal growth in all areas of the small intestine, with the greatest capacity for change in the distal bowel.

We next assessed whether this increase in presumably functional intestinal mucosa would result in increased serum citrulline in SERTKO animals. Serum citrulline is a non‐protein amino acid demonstrated in multiple reports to be a reliable biomarker for enterocyte mass (Crenn et al. 2008; Gutierrez et al. 2014). We analyzed serum samples from WT and SERTKO mice and found significant increases in serum citrulline in SERTKO mice, commensurate with overall changes in VH in SERTKO mice. This suggests that the histologic findings of enhanced mucosal growth correlate to an *in vivo* increase in total enterocyte mass, and support the concept of enhanced 5‐HT signaling as a therapeutic target to add new enterocytes and thus potentially functional intestinal mucosa. Further analysis was thus directed at determining absorptive function of these mice.

To evaluate for absorption, we utilized a previously described method of isolated intestinal segments to measure carbohydrate and peptide absorption in SERTKO mice compared to WT (Sullins et al. 2014). In our study we analyzed for the absorption of glucose and a fluorescent‐tagged peptide D‐Ala‐Lys‐AMCA, and found significant increases for both substrates in SERTKO mice compared to WT. We chose D‐Ala‐Lys‐AMCA as a surrogate for peptide absorption as it is a dipeptide which has been shown to be a specific substrate of the PEPT1 transporter in the small intestine (Groneberg et al. 2001). We now know from other experiments in our laboratory that, in the intact mouse, carbohydrate, and fat absorption are increased in the setting of enhanced serotonin signaling (Park et al. 2019), and since we have shown differences in how the proximal and distal small intestine respond to serotonin, here we sought to evaluate glucose and protein absorption in isolated segments rather than the whole mouse. We analyzed absorption in the proximal, middle and distal small bowel, and while not all increases observed in the proximal regions met statistical significance, both substrates had significantly increased absorption in the distal small bowel. This is in agreement with our histologic findings that the greatest area of increase in mucosal growth also occurs in the distal bowel. While we feel our current findings suggest an increase in functional mucosa as a result of enhanced serotonin signaling, there is the possibility that the increased mucosal growth and the increased absorption are not directly related. The possibility exists that there is a direct effect of serotonin. For example, serotonin may directly affect organic cation transporters (OCTs) in the intestine which could alter mucosal absorption (Liang et al. 2015), and further study is warranted to clarify the exact mechanism of our current findings. Importantly, our current study found increased absorption in all regions of the bowel, providing novel evidence in support of enhanced 5‐HT signaling in the ENS as a valuable therapeutic target for adding functional enterocytes.

These data are the foundation for our planned experiments in clinically relevant settings. For example, we plan to investigate the utility of enhanced serotonin signaling on mucosal growth and absorption following small bowel resection, which is known to result in intestinal mucosal adaptation as well as changes in the microbiome (Tappenden 2014; Sommovilla et al. 2015). The underlying mechanisms to these changes remain incompletely understood, but we feel enteric serotonin signaling likely plays an important role. As previously mentioned, neuronal serotonin has been implicated as a regulator of intestinal mucosal growth (Gross et al. 2012), and more recent evidence suggests a link between the microbiome and serotonergic neural pathways (De Vadder et al. 2018). The involvement in a wide range of physiologic processes makes enteric serotonin signaling an attractive potential therapeutic target and key area of ongoing study, specifically in optimization of mucosal growth and function in disease states such as short bowel syndrome.

In conclusion, we have determined that mice with enhanced 5‐HT signaling have increased intestinal mucosal growth throughout the small bowel, and this growth results in increased serum markers for enterocyte mass as well as increased glucose and peptide absorption. These data are critical for supporting the concept of targeting the serotonin signaling system in the small bowel to stimulate the growth of functioning intestinal mucosa. In this setting, 5‐HT acts as an intestinal growth factor rather than a neurotransmitter, and further study is warranted to define the complex mechanisms involved in this signaling pathway with a broader goal of developing new therapies for the treatment of short bowel syndrome and intestinal insufficiency.

## Conflict of Interest

The authors have no conflict of interest to report.
